# Supplementary material for: Identification of photoperiod-induced specific miRNAs in the adrenal glands of Sunite sheep (Ovis aries)
Source: Front Vet Sci. 2022 Jul 22;9:888207. doi: 10.3389/fvets.2022.888207 (PMC9354845; doi:10.3389/fvets.2022.888207)
Supplement: Supplementary file 1 [file Data_Sheet_1.ZIP › Supplementary file/miRDeep2 file.zip!/miRDeep2 file/Know_Structure/SPLP42A2/oar-mir-495.pdf]

miRBase precursor : oar-mir-495
Total read count : 16319
oar-miR-495-5p read count : 131
oar-miR-495-3p read count : 16168
remaining reads : 20

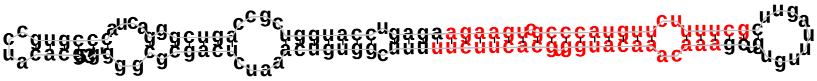

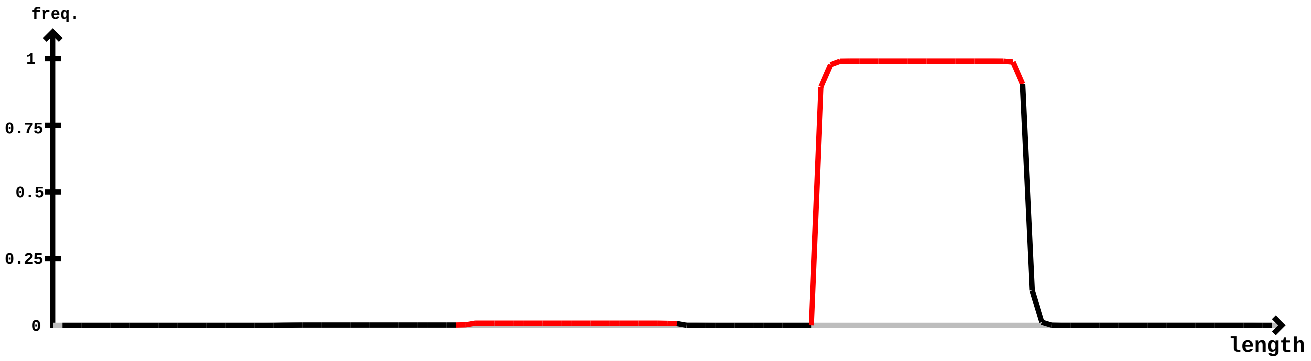


```
                                                                                            oar-miR-495-3p
                                                        oar-miR-495-5p
5'- gcacauccgugcccaucagggcugaccgcugguaccugagaagaagucgcccauguucuuuucgcuugauuugugucgaaacaaacauggugcacuucuuuuucggugucaaaucucagcgcgggg  -3'  exp
    ((((....))))((....(.(((((....(((((((.((((((((((.(((((((((..((((((.........).)))))..))))))).))))))))))))).))))))).....)))))).)..))  reads  mm
    .....................cugaccgcugguaccugagaa....................................................................................  1      0
    ......................ugaccgcugguaccugaga.....................................................................................  2      0
    ......................ugaccgcugguaccugagaa....................................................................................  6      0
    .......................gaccgcugguaccugagaa....................................................................................  6      0
    ........................accgcugguaccugagaa....................................................................................  2      0
    ........................accCcugguaccugagaa....................................................................................  1      1
    .........................................agaagucgcccauguucuuuu................................................................  4      0
    .........................................agaagucgcccauguucuuuuc...............................................................  4      0
    .........................................agaagucgcccauguucuuuucg..............................................................  7      0
    ..........................................gaagucgcccauguucuuuu................................................................  8      0
    ..........................................gaagucgcccauguucuuuuc...............................................................  8      0
    ..........................................gaagucgcccauguucuuuucg..............................................................  96     0
    ..........................................gaagucgcccauguucuuuucgcu............................................................  1      0
    ..........................................gaagucgcccauguucuuuucgcuu...........................................................  2      0
    ...........................................aagucgcccauguucuuuucgcu............................................................  1      0
    ..............................................................................aaacaaacauggugcacuu.............................  3      0
    ..............................................................................aaacaaacauggugcacuuc............................  48     0
    ..............................................................................aaacaaacauggugcacuucu...........................  1232   0
    ..............................................................................aaacaaacauggugcacuucuu..........................  11970  0
    ..............................................................................aaacaaacauggugcacuucuuu.........................  1311   0
    ..............................................................................aaacaaacauggugcacuucuuuu........................  31     0
    ...............................................................................aacaaacauggugcacuu.............................  1      0
    ...............................................................................aacaaacauggugcacuuc............................  4      0
    ...............................................................................aacaaacauggugcacuucu...........................  106    0
    ...............................................................................aacaaacauggugcacuucuu..........................  635    0
    ...............................................................................aacaaacauggugcacuucuuu.........................  568    0
    ...............................................................................aacaaacauggugcacuucuuuu........................  31     0
    ...............................................................................aacaaacauggugcacuucuuuuu.......................  2      0
    ................................................................................acaaacauggugcacuucu...........................  7      0
    ................................................................................acaaacauggugcacuucuu..........................  23     0
    ................................................................................acaaacauggugcacuucuuu.........................  71     0
    ................................................................................acaaacauggugcacuucuuuu........................  115    0
    ................................................................................acaaacauggugcacuucuuuuu.......................  3      0
    .................................................................................caaacauggugcacuucuuuu........................  3      0
```

```
                                                                                  oar-miR-495-3p
                                         oar-miR-495-5p
gcacauccgugcccaucagggcugaccgcugguaccugagaagaagucgcccauguucuuuucgcuugauuugugucgaaacaaacauggugcacuucuuuuucggugucaaaucucagcgcgggg

................................................................................caaacauggugcacuucuuuuu.......................     4          0
..................................................................................................uuucggugucaaaucucagcg.....     1          0
..................................................................................................uuucggugucaaaucucagcgcgg..     1          0
```
